# Impact of Diabetic Lesions on Pathology, Treatment, and Outcomes of Glomerular Diseases

**DOI:** 10.34067/KID.0000000000000247

**Published:** 2023-08-29

**Authors:** Young Ho Kim, Manish K. Saha, Yichun Hu, Srikar Kumar, Caroline J. Poulton, Susan L. Hogan, Patrick Nachman, J. Charles Jennette, Cynthia C. Nast, Amy K. Mottl

**Affiliations:** 1 University of North Carolina Kidney Center, UNC School of Medicine, Chapel Hill, North Carolina; 2 University of Minnesota Division of Nephrology and Hypertension, UM School of Medicine, Minneapolis, Minnesota; 3 Division of Nephropathology, Cedars-Sinai Medical Center, Los Angeles, California

**Keywords:** diabetes, glomerular disease, biopsy, pathology, ESKD, death, hospitalization

## Abstract

**Key Points:**

- People with glomerular disease (GD) and comorbid diabetes have similar baseline characteristics irrespective of superimposed diabetic lesions.
- Immunosuppression for GD with comorbid diabetes is the same regardless of superimposed diabetic glomerular lesions.
- ESKD or death is more rapid in GD and comorbid diabetes only in the presence of moderate-severe diabetic glomerular lesions.

**Background:**

We aimed to evaluate whether concomitant diabetic glomerulosclerosis (DGS) and its severity affect the treatment and outcomes of primary glomerular diseases (GDs) with comorbid diabetes.

**Methods:**

We conducted a retrospective review of people with diabetes and GD. We searched the GD Collaborative Network for biopsies from 2008 to 2015 among persons with diabetes and any of the following diagnoses: FSGS, IgA nephropathy, minimal change disease, membranous nephropathy, or antineutrophil cytoplasmic autoantibody GN. Data were abstracted from health records and histologic diabetic glomerular class scored. The primary composite end point was ESKD or death. Multivariable Cox regression models tested whether any or the severity of diabetes histopathology affected the primary end point.

**Results:**

Data from 134 cases were available for analysis (78 DGS+GD and 56 GD alone). Diabetes duration and glycemic control were similar between the two groups (*P* = 0.2; *P* = 0.09, respectively). Use of immunosuppression did not differ between the two groups (*P* = 0.3). The composite end point was significantly higher in DGS+GD (22.5 cases per 100 person-years [95% confidence interval (CI), 16.6 to 30.5]) versus GD alone (10.2 cases per 100 person-years [95% CI, 6.4 to 16.2]). Regression analyses demonstrated that compared with the GD-alone group, the risk for the composite end point was similar in the group with mild DGS+GD (DGS class 1, 2a) (hazard ratio, 1.15 [95% CI, 0.54 to 2.43]) while the group with severe DGS+GD (DGS class 2b, 3, 4) had a greater risk (hazard ratio, 3.60 [1.79 to 7.22]).

**Conclusions:**

Among people with diabetes and GD, mild diabetic glomerular lesions on biopsy do not affect outcomes, but moderate-severe lesions increase the risk for ESKD and death. Whether use of immunosuppression, particularly glucocorticoids, is less successful in inducing GD remission in people with moderate-severe diabetic lesions will be a focus of future study in a larger population.

## Graphical abstract

**Figure:**
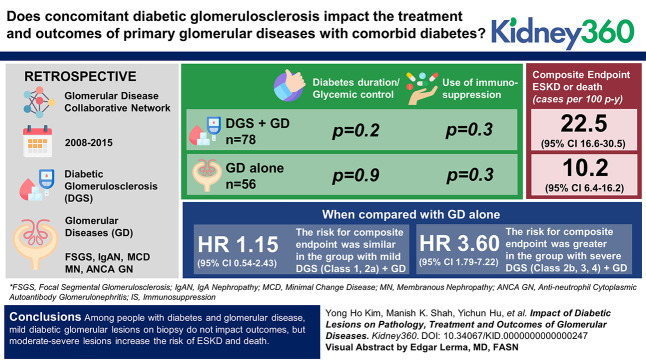

The prevalence of diabetes has grown to 14.6% of all US adults, a quarter of whom are unaware of their diagnosis.^1^ Minority populations have the highest rates of diabetes, prevalent in 18.3% of Black, 16.6% of Hispanic, and 16.4% of Asian populations.^1^ Given the rising prevalence of diabetes in the United States, it is not surprising that biopsies with diabetic kidney disease, with or without superimposed nondiabetic kidney disease, are also on the rise.^2,3^ Moreover, the prevalence of diabetes among patients with some glomerular diseases (GDs) may be even higher than in the general population (21.4%–45.7% versus 14.6%).^4^ Data from Kaiser Permanente showed a diabetes prevalence of 28% in FSGS (*n*=973) and 16.1% in membranous nephropathy (MN) (*n*=317) versus 13.9% in minimal change disease (MCD) (*n*=274) and 11.8% in IgA nephropathy.^4^ This heterogeneity is likely due to enrichment for diabetes risk factors such as higher rates of Black patients in the FSGS sample and older age among the FSGS, MN, and MCD strata (50–52 years) versus IgA nephropathy stratum (43 years).

The largest single-center study of clinicopathologic findings in patients with diabetes was performed at Columbia University, where they found that 620 of 2642 (23.5%) kidney biopsies performed in 2011 were from patients with diabetes.^5^ GD was diagnosed in approximately 40% of the cases in this series, either with or without concomitant diabetic glomerulosclerosis (DGS). These data underscore the high prevalence of not only a clinical diagnosis of diabetes in those undergoing kidney biopsy but also DGS comorbid with primary GD.

Despite the fact that diabetes is common among patients with GD, there has been little effort to explore it as a modifying factor in the pathogenesis, natural history, and treatment of GDs.^6,7^ People with diabetes are frequently excluded from clinical trials and observational cohort studies focused on GD.^8–11^ Diabetes is a systemic illness which can affect immunologic and inflammatory modulators,^12^ thus affecting potential for response to treatments, infectious^13–15^ and cardiovascular complications,^16,17^ and progression of kidney disease.^16,18^ Moreover, several lesions are shared between DGS and other GDs (*e.g.*, mesangial matrix expansion, podocyte activation and effacement, focal glomerulosclerosis, and tubulointerstitial and vascular lesions), potentially complicating etiologic interpretation of these lesions and clinical decisions regarding immunosuppression. One study did analyze data from people with GD, with versus without diabetes, and found less use of immunosuppression and more rapid progression to ESKD among those with diabetes but did not provide details on the severity of concomitant diabetic glomerular lesions.^16^ In this study, we aimed to describe the clinical and pathologic characteristics and longitudinal outcomes of persons with diabetes and GDs: IgA nephropathy, FSGS, MCD, MN, and antineutrophil cytoplasmic autoantibody-associated GN (ANCA GN). We hypothesized that patients with GD and diabetes, but without severe DGS, have similar kidney outcomes as patients without any DGS.

## Methods

We conducted a retrospective, observational study of a single-center cohort of people with diabetes and GD. This study was approved by the University of North Carolina Institutional Review Board and is in compliance with the Declaration of Helsinki.

### Population

**Figure 1:**
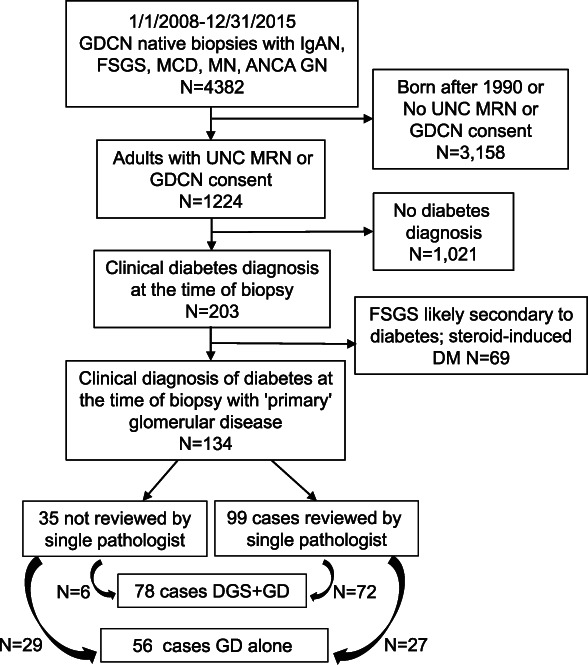
**Flow chart of selection criteria from the GDCN database for chart and biopsy review of biopsies from individuals with diabetes and one of the following GDs: IgA nephropathy, FSGS, MCD, MN, or ANCA GN.** Slides reviewed by a single pathologist included those with definite or questionable DGS based on the finding of variable GBM thickening in the biopsy report. There were five cases with definite DGS that were not reviewed by the pathologist because of unavailability of slides. These were scored based on the biopsy report findings, and all five had either severe mesangial expansion (class 2b) or KW nodules (class 3). ANCA GN, antineutrophil cytoplasmic autoantibody GN; DGS, diabetic glomerulosclerosis; GBM, glomerular basement membrane; GD, glomerular disease; GDCN, Glomerular Disease Collaborative Network, KW, Kimmelstiel Wilson; MCD, minimal change disease; MN, membranous nephropathy.

The GD Collaborative Network (GDCN) at the University of North Carolina (UNC) comprises more than 300 participating clinics and academic sites predominantly located in the southeastern United States. The GDCN database was searched between January 1, 2008, and December 31, 2015, for clinical kidney biopsies with the following pathologic diagnoses: IgA nephropathy, FSGS, MCD, MN, or ANCA GD. Individuals with a long-term GDCN consent for review or a UNC medical record number were approved for review by the UNC institutional review board. Charts were manually reviewed to identify patients with diabetes (Y.H.K., M.K.S., S.K., and A.K.M.). Patients were included as participants in this study if they had type 1 or type 2 diabetes diagnosed before their first native kidney biopsy, at least ten nonsclerotic glomeruli per section on biopsy, and at least 1-year follow-up. Exclusion criteria included steroid-induced diabetes, solitary kidney, concurrent autoimmune disease or major systemic disease (*e.g.*, cystic fibrosis, advanced heart failure, history of any transplant), or immunosuppressive therapy at the time of biopsy for extrarenal indication. Biopsies with FSGS specified on the clinical biopsy report as secondary to DGS were also excluded, as were cases in which the reviewing pathologist (C.C.N.) felt there was diabetic pathology out of proportion to the degree of FSGS (Figure 1).

### Data Collection

Sociodemographic and clinical data were extracted from paper and electronic health records. Baseline data were defined as those captured at or near (within 3 months) the time of kidney biopsy. Treatment categories included the use of glucocorticoids, mycophenolate or azathioprine, calcineurin inhibitors, rituximab, cyclophosphamide, or others. Creatinine values were extracted at 3-month intervals, as available. Hospitalizations, ESKD, and death were adjudicated by a single reviewer using available health records (Y.H.K., M.K.S., S.K., and A.K.M.). ESKD was defined as initiation of dialysis or kidney transplantation.

DGS was either noted as present on the clinical nephropathology report or as questionable if there was mild diabetic pathology identified as variable glomerular basement membrane thickening on the report. A pathologist (C.C.N.) then reviewed the light microscopy slides and electron micrographs for all biopsies with the diabetic pathology as present or questionable to generate a diabetes glomerular class as per the Renal Pathology Society.^19^ All other pathology data were extracted from clinical nephropathology reports for all participants. FSGS lesion subtype (tip lesion, perihilar, collapsing, or not otherwise specified) was recorded. For IgA nephropathy and ANCA GN, percent crescents, crescent type (cellular/fibrocellular versus fibrous), and segmental sclerosis were collected. For IgA nephropathy, the presence of mesangial and/or endocapillary hypercellularity was also noted. MEST-C scores were not available for most participants, so they were not included for analysis. Parameters that were extracted and scored for all biopsies as none, mild, moderate, or severe included acute tubular injury, arteriosclerosis (where available), and interstitial fibrosis and tubular atrophy (IFTA). The category GD+DGS was assigned when individuals with GD also had evidence of DGS on kidney biopsy. All others were categorized as GD alone.

### Statistical Analyses

Demographic, clinical, and pathologic characteristics and outcomes were quantified using percentages for categorical variables and median and interquartile ranges for continuous variables. eGFR was calculated using the CKD Epidemiology Collaboration equation (including race adjustment).^20^ Characteristics were compared between groups with and without superimposed DGS, with *P* values calculated by the Fisher exact test for categorical variables and the Kruskal-Wallis test for continuous variables. Owing to small sample sizes, comparison tests were not performed within each GD subtype.

Rates of hospitalization or ESKD/death were calculated using the number of first events divided by follow-up time standardized to 100 person-years (p-y). The kidney biopsy date was used as the start date, and patients who did not reach an end point were censored at the last available follow-up date. Kaplan-Meier curves were modeled using a composite end point of ESKD or death per 100 p-y for: (*1*) DGS+GD versus GD alone and (*2*) no DGS versus mild DGS (glomerular class 1, 2a) versus moderate and severe DGS (glomerular classes 2b, 3, and 4).

Multivariable Cox regression modeling was used to measure the effect of DGS and its severity on the risk for ESKD or death with hazard ratios (HRs), 95% confidence intervals (CIs), and *P* values reported. A priori specified covariates included age group (<40 versus 40–60 versus >60 years), sex, race/ethnicity (Black, Latinx, or White), and glomerulonephropathy type. There was insufficient power to include additional clinical variables. Post hoc univariate Cox regression analyses were performed to determine whether Black race/Hispanic ethnicity or the collapsing variant of FSGS was associated with time to ESKD/death in the FSGS subcohort.

## Results

Native kidney biopsies performed during the specified period and with a diagnosis of FSGS, IgA nephropathy, MCD, MN, or ANCA GN included 4382 individuals (Figure 1). Of the 1224 eligible for chart review, 203 (16.6%) had a clinical diagnosis of diabetes. We excluded 69 who were deemed to have steroid-induced diabetes and/or FSGS secondary to diabetes due to severe or nodular glomerulosclerosis disproportionate to the degree of FSGS, leaving 134 individuals (10.9% of the original 1224) with diabetes and a primary GD available for analyses. There were 78 patients in the DGS+GD group and 56 individuals in the GD-alone group. Of the 99 biopsies with definite or questionable DGS reviewed, 72 (73%) had at least class 1 DGS. Among the 72 individuals, diabetic glomerular class was assigned by the study pathologist as follows: 1, 2a, 2b, 3, and 4 for 19 (27%), 14 (20%), 7 (10%), 27 (37%), and 5 (7%) individuals, respectively. There were an additional six cases for whom slides could not be retrieved, and all of them had definite DGS, and diabetic glomerular class was assigned according to the biopsy report as follows: those with severe mesangial expansion but without Kimmelstiel Wilson nodules (*n*=1) were assigned class 2b and those with Kimmelstiel Wilson nodules were assigned class 3 (*n*=5).

Sociodemographic characteristics, diabetes type, hemoglobin A1c, and diabetes duration were statistically similar between the two groups (Table 1). The DGS+GD group were more often treated with insulin, had a higher urine protein:creatinine ratio, higher BP, and lower serum albumin than the GD-alone group.

**Table 1:** Cross-sectional sociodemographic and clinical data collected within 3 months of initial kidney biopsy in 134 participants with diabetes and either FSGS, minimal change disease, membranous nephropathy, IgA nephropathy, or antineutrophil cytoplasmic autoantibody GN, stratified by the presence of diabetic glomerulosclerosis^a^ in the Glomerular Disease Collaborative Network

Sociodemographic and Clinical Characteristic	No. Missing	DGS+GD *N*=78 Median (IQR)	GD Alone *N*=56 Median (IQR)	*P* Value
Age at time of biopsy, yr	0	60 (49–68)	60 (48–69)	0.5
Female sex, *No.* (%)	0	33 (42)	28 (50)	0.4
**Race/ethnicity, *No.* (%)**				
Black	8	27 (36)	19 (37)	0.9
Hispanic		2 (3)	3 (6)	
Non Hispanic White		44 (59)	30 (58)	
Others		1 (1)	0 (0)	
**GD type, *No.* (%)**				
FSGS	0	49 (63)	23 (41)	0.04
IgA nephropathy		10 (13)	11 (20)	
MCD		4 (5)	1 (2)	
MN		8 (10)	8 (14)	
ANCA GD		7 (9)	13 (23)	
Type 2 diabetes, *No.* (%)	9	66 (90)	50 (96)	0.3
Diabetes duration at the time of biopsy, mo	59	45 (30–55)	51 (32–59)	0.2
HbA1c at the time of biopsy, %	65	7.2 (6.4–8.9)	6.3 (5.9–7.5)	0.09
**DM treatment at time of biopsy, *No.* (%)**				
Insulin	0	43 (55)	12 (21)	0.007
Oral agents alone		28 (36)	32 (57)	
Diet controlled		7 (9)	12 (21)	
RASi treatment, *No.* (%)	26	47 (69)	28 (70)	1.0
Body Mass Index	29	32 (28–39)	33 (29–37)	0.7
Systolic Blood Pressure, mm Hg	18	143 (132–159)	135 (125–146)	0.02
Diastolic Blood Pressure, mm Hg	18	80 (72–84)	78 (70–81)	0.1
UPC at the time of biopsy, g/g	7	6.6 (3.0–12.0)	3.0 (1.6–5.8)	0.006
Creatinine at the time of biopsy, mg/dl	2	2.3 (1.5–3.4)	2.0 (1.2–2.9)	0.08
eGFR^b^ at the time of biopsy, ml/min per 1.73 m^2^	2	28 (17–50)	36 (21–69)	0.2
Serum albumin, mg/dl	29	2.9 (2.3–3.5)	3.4 (2.8–3.8)	0.02
Follow-up time, yr	0	2.0 (0.4–3.3)	2.9 (1.7–4.5)	0.008
Immunosuppression (ever)	0	67 (86%)	52 (93%)	0.3

DGS, diabetic glomerulosclerosis; GD, glomerular disease; IQR, interquartile range; MCD, minimal change disease; MN, membranous nephropathy; ANCA, antineutrophil cytoplasmic autoantibody; HbA1c, hemoglobin A1c; RASi, renin-angiotensin system iinhibitor; UPC, urine protein creatinine.

a Diabetic glomerulosclerosis defined as diabetes glomerular class 1–4 as defined by the Renal Pathology Society.

b eGFR calculated by the CKD-EPI_SCr_ equation using race-based adjustment.

Treatment approaches within each GD category were statistically similar between those with DGS+GD versus GD alone, although numerically patients with FSGS and DGS were less often treated with immunosuppression than those with FSGS alone (86% versus 93%; *P* = 0.3). All participants with MN and ANCA GD received immunosuppression (including steroids), regardless of the presence of DGS on pathology; however, patients with ANCA+DGS were treated less often with rituximab than those with ANCA GD alone (14% versus 46%).

Differences in kidney pathology parameters between the DGS+GD versus GD-alone groups included worse global glomerulosclerosis (*P* = 0.04), arteriosclerosis (*P* = 0.02), and IFTA (*P* = 0.001) in the former, but there was no difference in the presence of acute tubular injury (Table 2). Diabetes glomerular class was highly correlated with the severity of IFTA (*P* < 0.0001), arteriosclerosis (*P* = 0.002), systolic blood pressure (*P* = 0.0002), and urine protein creatinine (*P* < 0.0001), but only IFTA was associated with eGFR at the time of biopsy (*P* < 0.0001) (Supplemental Table 1, A–C).

**Table 2:** Severity of vascular and tubulointerstitial lesions in 134 participants with diabetes and FSGS, minimal change disease, MN, IgA nephropathy, or antineutrophil cytoplasmic autoantibody GN, stratified by the presence of diabetic glomerulosclerosis in the Glomerular Disease Collaborative Network

Histologic Index	No. Missing	DGS+GD *N*=78 *N* (%)	GD Alone *N*=56 *N* (%)	*P* Value
**ATI**				
None	1	53 (68)	37 (67)	0.2
Mild		11 (14)	13 (24)	
Moderate/severe		14 (18)	5 (9)	
Percent global glomerulosclerosis per biopsy, median (IQR)	9	26 (13–14)	15 (7–38)	0.04
**Arteriosclerosis**				
None	17	3 (4)	7 (15)	0.02
Mild		13 (19)	15 (32)	
Moderate/severe		54 (77)	25 (53)	
**IFTA**				
Mild	0	22 (28)	32 (57)	0.001
Moderate/severe		56 (72)	24 (43)	

DGS, diabetic glomerulosclerosis; GD, glomerular disease; ATI, acute tubular injury; IQR, interquartile range; IFTA, interstitial fibrosis and tubular atrophy.

Collapsing FSGS features occurred in 20% of the DGS+GD group versus 14% in the GD-alone group (*P* = 0.4). Crescentic IgA nephropathy was numerically more common among the IgA nephropathy-alone group (36%) compared with the DGS+IgA nephropathy group (10%), although this difference was not statistically significant (*P* = 0.2). There was also a statistically nonsignificant but numeric trend of a greater proportion with endocapillary and mesangial hypercellularity in the IgA nephropathy-alone group and of segmental sclerosis in the DGS+IgA nephropathy group (Supplemental Table 2). Among the ANCA GD group, crescentic disease was similar regardless of concurrent DGS; however, the percent fibrous crescents was 67% in DGS+GD versus 20% in the GD-alone group (*P* value = 0.05) (Supplemental Table 2).

The rate of hospitalization in the full cohort was 35 (95% CI, 29 to 41) per 100 p-y, with similar rates between those with DGS+GD versus GD alone, 34 (95% CI, 27 to 43) and 35 (95% CI, 27 to 44) hospitalizations per 100 p-y, respectively. When stratified by GD subtype, hospitalization rates differed according to superimposed DGS only in the FSGS cohort, 37 (95% CI, 26 to 51) per 100 p-y in the DGS+FSGS group versus 17 (95% CI, 9 to 31) hospitalizations per 100 p-y in the FSGS-alone group.

The incidence rate of ESKD or death was 17 (95% CI, 13 to 21) per 100 p-y in the full group but significantly higher in the group with DGS+GD (22.5 [95% CI, 16.6 to 30.5] per 100 p-y) versus GD alone (10.2 [95% CI, 6.4 to 16.2] per 100 p-y). When stratified by GD subtype, incident ESKD or death rates differed according to superimposed DGS only in the FSGS cohort, with DGS+FSGS: 36 (95% CI, 25 to 51) versus FSGS alone: 12 (95% CI, 6 to 25) per 100 p-y. The results of hospitalization and ESKD/death analyses according to the GD subgroup are available in Supplemental Table 3, A and B. Post hoc univariate regression analyses restricted to the FSGS subgroup found that neither Black race/Hispanic ethnicity nor the collapsing variant was associated with time to ESKD or death: HR 1.25 (95% CI, 0.65 to 2.40) and 2.01 (95% CI, 0.86 to 4.67), respectively.

**Figure 2:**
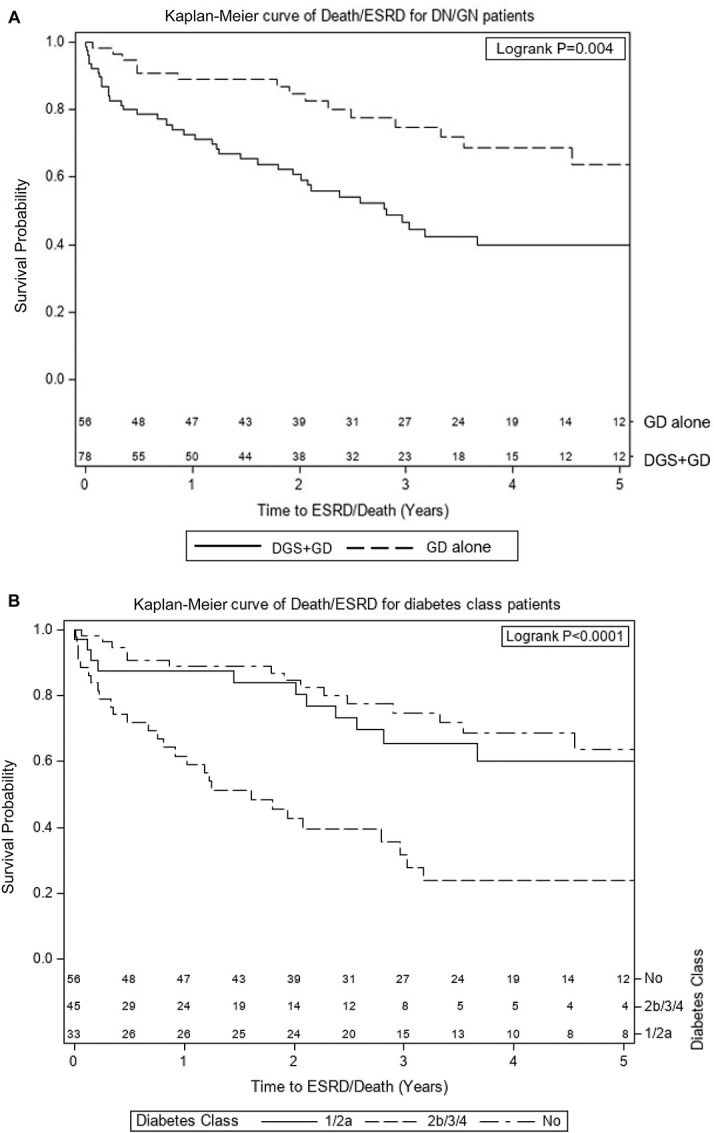
**Among people with diabetes at the time of glomerular disease diagnosis, time to ESKD or death is more rapid only in the groups with diabetic glomerular class 2b, 3 or 4).** (A and B) Kaplan-Meier curves of time to ESKD or death from time of biopsy among patients with diabetes and one of the following GDs: FSGS, MCD, IgA nephropathy, or ANCA GN, stratified by (A) the presence of DGS or (B) diabetes glomerular class where class 0=no diabetic glomerulopathy and classes 1–4 are per the International Society of Nephrology diabetes glomerular classification system (*N*=134). ANCA GN, antineutrophil cytoplasmic autoantibody GN; DGS, diabetic glomerulosclerosis; GD, glomerular disease; MCD, minimal change disease.

Kaplan-Meier analysis of time to ESKD or death (Figure 2A) demonstrated more rapid progression in participants with DGS+GD versus GD alone (*P* = 0.004). After reclassifying DGS+GD according to severity of diabetes glomerular class (Figure 2B), time to ESKD or death in the stratum with mild DGS (class 1 or 2a) was similar to the stratum with GD alone, whereas the stratum with severe DGS (class 2b, 3, or 4) was significantly more rapid (*P* < 0.0001).

Multivariable Cox regression analyses of time to ESKD/death, incorporating age, sex, Black/Hispanic race/ethnicity, GD type, and DGS pathology, identified DGS pathology as the only predictive covariate (HR 2.13 [95% CI, 1.19 to 3.82], Table 3). Similar Cox regression analyses substituting diabetes glomerular class for DGS found class 1/2a to be similar to those without DGS on pathology, whereas those with glomerular class 2b/3/4 had a significantly higher risk of ESKD or death: HR 3.95 (95% CI, 2.01 to 7.74). Black/Hispanic race/ethnicity, elevated in both models, was marginally significant in the model, including diabetes glomerular class (HR 1.79 [95% CI, 0.99 to 3.24]).

**Table 3:** Multivariate Cox regression models of time from kidney biopsy to ESKD or death, incorporating either any diabetic glomerular disease on pathology (model 1) or incorporating diabetes glomerular class on pathology (*N*=126)

Characteristic	Model 1 HR (95% CI)	Model 2 HR (95% CI)
**Age group, yr**		
<40	0.65 (0.26 to 1.58)	0.61 (0.25 to 1.52)
40–60	(0.66 to 2.19)	0.94 (0.51 to 1.74)
>60	Referent	Referent
Female sex	0.85 (0.47 to 1.52)	0.83 (0.46 to 1.47)
Black or Latinx race/ethnicity	1.74 (0.97 to 3.12)	1.79 (0.99 to 3.24)
**GD subtype**		
FSGS	1.79 (0.72 to 4.45)	1.36 (0.54 to 3.42)
MCD	0.89 (0.28 to 2.83)	0.18 (0.02 to 1.56)
MN	0.30 (0.04 to 2.59)	0.54 (0.16 to 1.88)
IgA nephropathy	0.57 (0.16 to 1.98)	1.07 (0.33 to 3.45)
ANCA	Referent	Referent
DGS on pathology	2.13 (1.19 to 3.82)	Not applicable
**Diabetes glomerular class**		
0	Not applicable	Referent
1/2a		1.14 (0.54 to 2.41)
2b/3/4		3.95 (2.01 to 7.74)

HR, hazard ratio; CI, confidence interval; GD, glomerular disease; MCD, minimal change disease; MN, membranous nephropathy; ANCA, antineutrophil cytoplasmic autoantibody; DGS, diabetic glomerulosclerosis.

## Discussion

This study comprises a large, detailed analysis of a real-world cohort of patients with GD in the setting of diabetes and the impact of concomitant DGS of varying severity on the composite outcome of death or ESKD. Among people with biopsy proven primary GD, 16.6% had diabetes. After excluding steroid-induced diabetes and focal segmental glomerular scarring secondary to severe DGS as concurrent FSGS, the prevalence rate was reduced to 10.9%, similar to the prevalence of diabetes in the general population of North Carolina during the same time periods as biopsies occurred.^21^ The finding that the prevalence of diabetes in primary GD is similar to the general population is in contrast to a large dataset compiled from Kaiser Permanente,^4^ possibly due to our exclusion of individuals with focal segmental glomerular scarring secondary to severe DGS. However, distinguishing primary from secondary FSGS is extremely difficult, and it is possible that our estimates are somewhat conservative. Moreover, it may be that people with diabetes are less commonly biopsied, also potentially leading to an underestimate of the true prevalence of GD in diabetes.

We also found that the sociodemographic and clinical characteristics of the group with DGS+GD were similar to those with GD alone, with the primary exceptions of a greater frequency of insulin therapy and higher baseline proteinuria in the former group. The similarities between these groups underscore the difficulty of postulating the underlying glomerular pathology without a kidney biopsy in persons with diabetes.

IFTA was more severe in the DGS+GD versus GD-alone group, but the difference in arteriosclerosis was of marginal significance. While worse indices of scarring may be due to diabetic pathology, it is also possible that the patients with DGS+GD are biopsied later in the process of GD pathology rather than having significant DGS before the onset of GD. We tried to address this question by examining the severity of disease activity versus chronicity in those with IgA nephropathy and ANCA GN. Although there were no statistically significant differences, there were numeric trends for more chronicity in the DGS+GD versus GD-alone group. Among the IgA nephropathy group, there was a trend for a greater proportion with crescentic disease and mesangial and endocapillary hypercellularity among those with IgA nephropathy alone but more segmental sclerosis among those with DGS+IgA nephropathy. Similarly, among the ANCA GN group, there was a greater proportion with cellular/fibrocellular crescents in the ANCA GN stratum as opposed to fibrous crescents in the DGS+ANCA GN stratum. These findings warrant further study with larger sample sizes to determine whether some people with DGS+GD are biopsied unnecessarily late in the disease course.

Immunosuppression use did not differ between the two groups; however, those with DGS+GD suffered a much higher rate of the composite end point of ESKD and death. When DGS was modeled by severity of diabetic glomerular class, those with class I or 2a had similar rates to the group without DGS, confirming results from studies of all causes of nondiabetic kidney disease.^22^ Among those with diabetes glomerular class 2b or worse, 40% suffered ESKD or death in 1 year and 60% in 2 years. Such prognostic implications are important to discuss with patients, particularly during shared decision making regarding immunosuppression. Whether immunosuppression is less likely to induce disease remission in people with GD and superimposed moderate-to-severe diabetic glomerular lesions will require a larger population of study.

FSGS was the largest GD subgroup, which may have been the reason it was the only one to identify a difference in the composite end point ESKD or death according to the presence of DGS. Other postulated explanatory factors include greater representation of the collapsing variant of FSGS or African ancestry (with possible apolipoprotein 1 associated disease), both of which carry a worse clinical prognosis. Post hoc analyses showed a statistically nonsignificant relationship between these two factors and the composite end point, so further exploratory analyses were not undertaken. Notably, however, the point estimates were suggestive of a possible effect, and further studies should be undertaken in a larger sample size. This is particularly important given that the collapsing variant (also termed extraglomerular hypercellularity) has been found to be more common in people with diabetes and DGS.^23,24^

To the best of our knowledge, other than case reports, similar studies investigating DGS as a disease modifier among patients with these specific GDs are lacking. One study compared outcomes of nine individuals with IgA nephropathy+DGS with 27 with DGS alone and reported that after a follow-up of 31 months, while there was a similar rate of decline in creatinine clearance, there was a higher frequency of ESKD in the group with DGS alone (*P*, 0.04).^25^ A case series of 23 individuals with postinfectious GN superimposed on DGS found that nearly half required dialysis at presentation, and at a mean follow-up of 14.6 months, 75% had died or reached ESKD.^26^ Notably, 30% had nodular glomerulosclerosis and an additional 39% had moderate and 8.7% had severe glomerulosclerosis. Alternatively, a Danish study of patients hospitalized with ANCA-associated vasculitis (including individuals without kidney manifestations) found that diabetes did not affect overall survival, but CKD at discharge presented an increased absolute risk ratio of 2.6 (95% CI, 2.1 to 3.2).^27^

Lim *et al.* (2021) compared 153 people with diabetes and GD with 448 people with GD alone, wherein the predominant GD subtypes were MCD, FSGS, IgA nephropathy, ANCA GN, and lupus nephritis while 15% had other diagnoses.^16^ Individuals with diabetes were older and had worse baseline clinical parameters, including higher systolic BP, lipids and cardiovascular disease history, greater proteinuria, and lower eGFR. There was less use of immunosuppression in people with (56%) versus without (72%) diabetes, and although there was also less frequent use of glucocorticoids, the peak daily dose and duration did not differ. Rates of ESKD and cardiovascular-related hospitalization were higher among those with diabetes; however, there was no difference in mortality according to diabetes status. Contrary to our findings, concurrent histologic DGS was not a significant predictor of ESKD in multivariable models, but little detail is provided regarding the severity of DGS.^16^

There are weaknesses in this study, including a relatively small sample size, particularly when stratifying by GD subtype, as well as a fair amount of missing data with which to determine the duration and severity of diabetes. We did not investigate extraglomerular effects of diabetes, specifically arteriolar hyalinosis, which has been identified as prognostic of general CKD progression.^28^ The strengths of our study include the extensive clinical and histopathologic detail available for analysis, in conjunction with assignment of diabetes glomerular class by a nephropathologist.

We have confirmed that among individuals with DGS+GD but mild diabetic glomerular pathology (class 1 and 2a) rates of death and ESKD are similar to those with GD alone. This finding is important for providing prognostic information to people with diabetes and GD and may be important in clinical decision making. We also have raised the possibility that some people with diabetes may not be biopsied sufficiently early, as demonstrated in the IgA nephropathy and ANCA GD subgroups, who demonstrated greater chronicity and less activity, in the subgroup with DGS+GD versus GD alone. This practice may lead to later identification of an underlying primary GD, potentially decreasing the opportunity for response to treatment. Further research is indicated, inclusive of people with GD with and without diabetes, and in larger, prospective studies, to better understand the clinical, histopathologic and long-term consequences of diabetes in people with GD.

## Supplementary Material

SUPPLEMENTARY MATERIAL

## Data Availability

All data are included in the manuscript and/or supporting information.

## Supplemental Material

Supplemental Table 1A. Cross-sectional histologic and clinical data collected within 3 months of initial kidney biopsy in 134 participants with diabetes and either FSGS, MCD, MN, IgAN, or ANCA GN, stratified by the severity of diabetic glomerulosclerosis in the Glomerular Disease Collaborative Network.

Supplemental Table 1B. Cross-sectional histologic and clinical data collected within 3 months of initial kidney biopsy in 134 participants with diabetes and either FSGS, MCD, MN, IgAN, or ANCA GN, stratified by the severity of interstitial fibrosis and tubular atrophy (IFTA) in the Glomerular Disease Collaborative Network.

Supplemental Table 1C. Cross-sectional histologic and clinical data collected within 3 months of initial kidney biopsy in 117 participants with diabetes and either FSGS, MCD, MN, IgAN, or ANCA GN, stratified by the severity of arteriosclerosis (missing *N*=17) in the Glomerular Disease Collaborative Network.

Supplemental Table 2. Distribution of glomerular histopathology characteristics among individuals with IgA nephropathy (IgAN) and antineutrophil cytoplasmic antibody GN (ANCA GN), stratified by the presence of superimposed DGS.

Supplemental Table 3A. Clinical outcomes of 41 individuals with diabetes and glomerular disease (GD) with versus without concurrent diabetic glomerulosclerosis (DGS), stratified by GN category of IgA nephropathy (IgAN) and antineutrophil cytoplasmic antibody (ANCA) pauci-immune glomerulonephritis (PIGD).

Supplemental Table 3B. Clinical outcomes of 83 individuals with diabetes and glomerular disease with versus without concurrent diabetic glomerulosclerosis (DGS), stratified by glomerular disease subtype.
