# Supplementary material for: Impact of Diabetic Lesions on Pathology, Treatment, and Outcomes of Glomerular Diseases
Source: Kidney360. 2023 Aug 29;4(10):1445–53. doi: 10.34067/KID.0000000000000247 (PMC10615380; doi:10.34067/KID.0000000000000247)
Supplement: SUPPLEMENTARY MATERIAL [file kidney360-4-01445-s001.docx]

Supplemental Table 1A. Cross-sectional histologic and clinical data collected within 3 months of initial kidney biopsy in 134 participants with diabetes and either FSGS, MCD, MN, IgAN or ANCA GN, stratified by the severity of diabetic glomerulosclerosis in the Glomerular Disease Collaborative Network.

|  | Missing N | DGS Class 0 | DGS Class 1/2a | DGS Class 2b/3/4 | P value* |
| --- | --- | --- | --- | --- | --- |
|  |  | N=56 | N=33 | N=45 |  |
| IFTA, N (%) | 0 |  |  |  | <0.0001 |
| None/mild |  | 33 (58%) | 18 (55%) | 4 (9%) |  |
| Moderate/severe |  | 23 (41%) | 15 (45%) | 41 (91%) |  |
| Arteriosclerosis, N (%) | 17 |  |  |  | 0.0002 |
| None/mild |  | 22 (47%) | 12 (41%) | 4 (10%) |  |
| Moderate/severe |  | 25 (53%) | 17 (59%) | 37 (90%) |  |
| RASi, N (%) | 26 | 28 (70%) | 18 (67%) | 29 (71%) | 0.9 |
| SBP, mmHg | 18 | 135 (125, 146) | 137 (122, 148) | 146 (140, 162) | 0.0002 |
| DBP, mmHg | 18 | 78 (70, 81) | 78 (70, 82) | 80 (73, 86) | 0.1 |
| HbA1c, % | 65 | 6.3 (5.9, 7.5) | 6.7 (6.1, 7.6) | 7.5(6.6, 9.8) | 0.1 |
| eGFR at biopsy, ml/min/1.73m2 | 2 | 36 (21, 69.22) | 31 (18, 55) | 27(17, 47) | 0.2 |
| UPCR at biopsy, gm/gm | 7 | 3.0 (1.6, 5.8) | 4.8 (1.7, 8.3) | 8.1 (4.4, 14.5) | <0.0001 |

*P value was calculated by Fisher Exact test for categorical variables and Kruskal-Wallis Test for continuous variables.

Supplemental Table 1B. Cross-sectional histologic and clinical data collected within 3 months of initial kidney biopsy in 134 participants with diabetes and either FSGS, MCD, MN, IgAN or ANCA GN, stratified by the severity of interstitial fibrosis and tubular atrophy (IFTA) in the Glomerular Disease Collaborative Network.

|  | Missing N | Absent/Mild IFTA | Moderate/Severe IFTA | P value* |
| --- | --- | --- | --- | --- |
|  |  | N=55 | N=79 |  |
| DGS Class, N (%) | 0 |  |  | <0.0001 |
| 0 |  | 33 (60%) | 23 (29%) |  |
| 1/2a |  | 18 (33%) | 15 (19%) |  |
| 2b/3/4 |  | 4 (7%) | 41 (52%) |  |
| Absent/Mild Arteriosclerosis, N (%) | 17 |  |  | <0.0001 |
| Absent/Mild |  | 27 (58%) | 11 (16%) |  |
| Mod/Severe |  | 20 (43%) | 59 (84%) |  |
| RASi, N (%) | 26 | 29 (69%) | 46 (70%) | 1.0 |
| SBP, mmHg | 18 | 134 (124, 144) | 145 (130, 158) | 0.003 |
| DBP, mmHg | 18 | 78 (72, 82) | 80 (70, 84) | 1.0 |
| HbA1c, % | 65 | 6.7 (6.0, 8.0) | 7.1 (6.3, 7.9) | 0.6 |
| eGFR at time of biopsy, ml/min/1.73m2 | 2 | 47.3 (28.7, 83.6) | 23 (15, 36) | <0.0001 |
| UPC at time of biopsy, gm/gm | 7 | 3.5 (1.6, 6.0) | 6.5 (2.8, 11.1) | 0.005 |

*P value was calculated by Fisher Exact test for categorical variables and Wilcoxon Two samples test for continuous variables

Supplemental Table 1C. Cross-sectional histologic and clinical data collected within 3 months of initial kidney biopsy in 117 participants with diabetes and either FSGS, MCD, MN, IgAN or ANCA GN, stratified by the severity of arteriosclerosis (missing N=17) in the Glomerular Disease Collaborative Network.

|  | Missing  N | Absent/Mild Arteriosclerosis  N=38 | Moderate/Severe Arteriosclerosis  N=79 | P value* |
| --- | --- | --- | --- | --- |
|  |  |  |  |  |
| DGS Class, N (%) | 0 |  |  | 0.0002 |
| 0 |  | 22 (58%) | 25 (32%) |  |
| 1/2a |  | 12 (32%) | 17 (22%) |  |
| 2b/3/4 |  | 4 (10%) | 37 (47%) |  |
| Absent/Mild IFTA, N (%) | 0 |  |  | <0.0001 |
| None/mild |  | 27 (71%) | 20 (25%) |  |
| Moderate/severe |  | 11 (29%) | 59 (75%) |  |
| RASi, N (%) | 14 | 22 (73%) | 52 (71%) | 1.0 |
| SBP, mmHg | 13 | 133 (124, 150) | 142 (130, 157) | 0.1 |
| DBP, mmHg | 13 | 80 (72, 84) | 79 (70, 84) | 0.5 |
| HbA1c, % | 49 | 6.6 (5.4, 8.0) | 7.0 (6.1, 7.7) | 0.6 |
| eGFR at time of biopsy, ml/min/1.73m2 | 2 | 42 (19, 84) | 29 (17, 50) | 0.1 |
| UPC at time of biopsy, gm/gm | 6 | 4.2 (1.5, 6.8) | 6.5 (2.8, 12.0) | 0.02 |

*P value was calculated by Fisher Exact test for categorical variables and Wilcoxon Two samples test for continuous variables.

| Supplemental Table 2. Distribution of glomerular histopathology characteristics among individuals with IgA nephropathy (IgAN) and Antineutrophil Cytoplasmic Antibody glomerulonephritis (ANCA GN), stratified by the presence of superimposed DGS. | | | | |
| --- | --- | --- | --- | --- |
| Histologic Index | IgAN  N=21 | | ANCA GN  N=20 | |
|  | DGS + IgAN  N=10 | IgAN alone  N=11 | DGS + ANCA  N=7 | ANCA alone  N=13 |
| Mesangial Hypercellularity, N (%) | 6 (60%) | 9 (82%) | N/A | N/A |
| Endocapillary Hypercellularity, N (%) | 2 (20%) | 5 (45%) | N/A | N/A |
| Segmental Sclerosis, N (%) | 5 (50%) | 4 (36%) | 6 (86%) | 10 (77%) |
| Crescents, N (%) | 1 (10%) | 4 (36%) | 6 (86%) | 10 (77%) |
| Crescent type, N  Cellular/Fibrocellular  Fibrous | 1 (100%)  0 | 4 (100%)  0 | 2 (33%)  4 (67%) | 8 (80%)  2 (20%) |

| Supplemental Table 3A. Clinical outcomes of 41 individuals with diabetes and glomerular disease (GD) with versus without concurrent diabetic glomerulosclerosis (DGS), stratified by glomerulonephritis category of IgA Nephropathy (IgAN) and Antineutrophil Cytoplasmic antibody (ANCA) Pauci-immune glomerulonephritis (PIGD). | | | | | | | |
| --- | --- | --- | --- | --- | --- | --- | --- |
| Clinical Variable | IgAN  N=21 | | | ANCA GN  N=20 | | | |
|  | DGS+IgAN  N=11 | IgAN alone  N=10 | p-value | | DGS+ANCA  N=7 | ANCA alone  N=13 | p-value |
| Incident hospitalization, per 100 person-years (CI 95%) | 40  (25, 64) | 38  (21, 71) | 0.9 | | 50  (26, 96) | 60  (43, 84) | 0.6 |
| Incident RRT or death, per 100 person-years (CI 95%) | 9  (3, 25) | 15  (6, 25) | 0.5 | | 23  (9, 62) | 7  (3, 20) | 0.1 |

| Supplemental Table 3B. Clinical outcomes of 83 individuals with diabetes and glomerular disease with versus without concurrent diabetic glomerulosclerosis (DGS), stratified by glomerular disease subtype. | | | | | | | | | |
| --- | --- | --- | --- | --- | --- | --- | --- | --- | --- |
| Clinical Variable | Focal Segmental Glomerulosclerosis (FSGS)  N=72 | | | Minimal Change Disease (MCD)  N=5 | | | Membranous Nephropathy (MN)  N=16 | | |
|  | DGS+FSGS  N=49 | FSGS alone  N=23 | p-value | DGS+MCD  N=4 | MCD alone  N=1 | p-value | DGS+MN  N=8 | MN alone  N=8 | p-value |
| Incident hospitalization, per 100 person-years (CI 95%) | 37  (26,51) | 17  (9, 31) | 0.02 | 12  (3, 47) | 0 | N/A | 23  (11, 48) | 30  (14, 64) | 0.6 |
| Incident RRT or death, per 100 person-years (CI 95%) | 36  (25, 51) | 12  (6, 25) | 0.008 | 6  (0.8, 42) | 0 | N/A | 12  (4, 36) | 8  (2, 32) | 0.07 |
